# TB and HIV: how can we reduce mortality?

**DOI:** 10.7448/IAS.15.6.18074

**Published:** 2012-11-11

**Authors:** S Lawn

**Affiliations:** University of Cape Town, Cape Town, South Africa

## Abstract

Despite ART scale-up, tuberculosis (TB) remains a leading cause of HIV-related deaths worldwide and much of this disease may remain unascertained. In patients receiving ART, TB incidence is highest during the first few months of treatment (many cases of which were prevalent disease missed by baseline screening) and long-term rates remain several-fold higher than background. We identify three groups of patients starting ART for which different interventions are required to reduce TB-related deaths. First, diagnostic screening is needed in patients who have undiagnosed active TB so that timely anti-tuberculosis treatment can be started. This may be greatly facilitated by new diagnostic assays such as the Xpert MTB/RIF assay and a novel point-of-care urine test for lipoarabinomannan (LAM). Second, patients with a diagnosis of active TB need optimised case management, which includes early initiation of ART (with early timing now defined by randomised controlled trials), trimethoprim-sulphamethoxazole prophylaxis and treatment of co-morbidity. Third, in high TB burden settings, all remaining patients who are TB-free at enrolment have high ongoing risk of developing TB and require optimised immune recovery (with ART ideally started early in the course of HIV infection), isoniazid preventive therapy and infection control to reduce infection risk. Further specific measures are needed to address multi-drug resistant TB (MDR-TB) and there are now new promising developments in antimycobacterial agents. Finally, in high burden settings, scale-up of all these interventions requires nationally and locally tailored models of care that are patient-centred and provide integrated health care delivery for TB, HIV and other co-morbidities.

